# DFT investigation of the Si–As co-doped SrTiO_3_ perovskite for hydrogen evolution *via* water splitting under UV-vis irradiation

**DOI:** 10.1039/d6ra01829g

**Published:** 2026-07-25

**Authors:** Yahaya Saadu Itas, Mayeen Uddin Khandaker, Faiza Benabdallah

**Affiliations:** a Applied Physics and Radiation Technologies Group, CCDCU, Faculty of Engineering and Technology, Sunway University 47500 Bandar Sunway Selangor Malaysia yitas@sazu.edu.ng mayeenk@sunway.edu.my; b Department of Physics, Sa'adu Zungur University Gadau Nigeria; c Department of Physics, College of Science, Korea University 145 Anam-ro Seongbuk-gu Seoul 02841 Republic of Korea; d Miyan Research Institute, International University of Business Agriculture and Technology Dhaka 1230 Bangladesh; e Department of Industrial and Systems Engineering, College of Engineering, Princess Nourah bint Abdulrahman University P.O. Box 84428 Riyadh 11671 Saudi Arabia

## Abstract

This study uses density functional theory (DFT) calculations combined with molecular dynamics (MD) simulations to evaluate the water-splitting performance of the SrTiO_3_ perovskite improved through synergistic Si and As co-doping. While static DFT calculations capture only a single atomic configuration, MD simulations provide finite-temperature atomic ensembles, enabling realistic assessments of water adsorption, dissociation, and radical-formation processes. Pristine SrTiO_3_ shows pronounced thermal fluctuations, indicating weak thermal management, likely caused by limited generation of charge carriers and their rapid recombination. In contrast, the Si-doped, As-doped, and especially Si–As co-doped systems achieve faster thermal stabilization, suggesting enhanced charge-carrier dynamics and improved photocatalytic activity. Electronic analysis reveals significant band-gap narrowing in the doped structures due to the introduction of dopant-induced electronic states near the conduction band, increasing charge-carrier mobility. The band-gap trend—pure (2.12 eV) > As-doped (1.47 eV) > Si–As co-doped (1.50 eV) > Si-doped (1.28 eV)—extends absorption toward 570 nm, enabling visible-light-driven photocatalytic activity that is not exhibited by pristine SrTiO_3_. Overall, Si–As co-doping markedly enhances SrTiO_3_'s water-splitting capability.

## Abstract 2

This study uses density functional theory (DFT) calculations combined with molecular dynamics (MD) simulations to evaluate the water-splitting performance of the SrTiO_3_ perovskite improved through synergistic Si and As co-doping.

## Graphical abstract

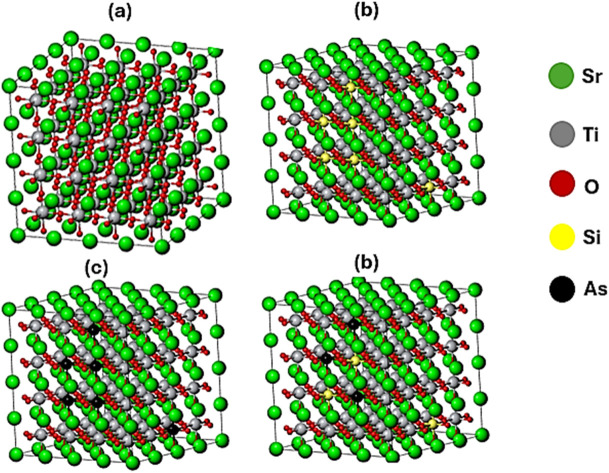

## 1 Introduction

Hydrogen, often referred to as green fuel, has recently become the most trending alternative to solve emission problems associated with fossil fuel decomposition.^1^ Hydrogen combustion produces water as a by-product.^2^ Although produced in relatively small quantities, modelled materials and methods can effectively enhance the large-scale production of hydrogen. Perovskite catalysts have recently gained significant attention in photocatalysis because they play critical roles in removing dyes and in wastewater treatment.^3^ Inspired by these applications, researchers are working hard to develop cost-effective ways to produce perovskite photocatalysts suitable for water splitting applications.^4^

Several works have been published on solar energy consumption, including its contribution to water splitting for hydrogen fuel production.^5,6^ Recently, scientists have converged on their efforts to develop cost-effective photocatalysts that are stable, non-toxic and efficient. The frequently explored photocatalysts include oxides of nitrogen, metal oxides and sulphides. For example, Ya-Nan *et al.* studied the water-splitting capacity of Co(OH)(NO_3_)·(CoNH) using atom substitution and vacancy creation.^7^ Their work uncovered that the optimized structure can be a highly efficient surface for photocatalytic water splitting. According to the research work by Yanet *et al.*, the photogeneration efficiency of Al_2_O_3_ significantly improved due to the addition of Ce^3+^/Ce^4+^ ions.^8^ However, the charge transport by metal oxides was found to be limited because of their low intrinsic electrical conductivity.^9^ They also demonstrate low diffusion due to highly dense crystal structures.^10^ Other reports have also revealed that metal oxide photocatalysts undergo significant phase transitions during cycling, which limit their performance.^11,12^

Recently, perovskites have attracted much attention due to their simple morphology and versatile chemical and physical properties. They can be engineered easily by doping to obtain improved electrical conductivity and photoconductivity.^13^ They also exhibit mechanisms that facilitate faster ion transport for efficient photoabsorption. Overall, perovskites have greater compositional flexibility, which allows improved redox and structural stability.^14^ To improve their performance, both metal and nonmetal doping approaches have been considered experimentally and theoretically.^15^ Research by Marcela *et al.* confirmed the successful doping of Ag atoms in a SrTiO_3_ photocatalyst for NO_*x*_ degradation.^16^ However, their findings failed to report a reliable way to annihilate toxicity due to lead effect. Another work by Jing *et al.* reported solvothermal synthesis of nitrogen-doped SrTiO_3_ with high visible light photocatalysis.^17^ On the other hand, the effects of metalloids such as arsenic (As), antimony (Sb), or boron (B) on the visible light and other optoelectronic properties of SrTiO_3_ have not been reported. Metalloids possess intermediate electronegativity with improved bonding behaviours. They have high tendency to introduce active sites and optimize the Seebeck coefficient, electrical conductivity, and thermal conductivity.^18^ For example, arsenic has been reported to effectively improve the HER efficiency of MoS_2_. Additionally, the p-type conductivity of graphene is improved significantly using boron doping.^19^ Other prominent properties of metalloids include long excitation energy and a tendency to form new active sites.

The present research work focuses on improving the water-splitting efficiency of SrTiO_3_*via* a nonmetal/metalloid co-doping strategy. Studies were conducted by comparing results obtained from separate Si and As doping against the Si–As co-doped SrTiO_3_ variant. The doping mechanism is designed to redistribute charges on Si, As and Si/As atomic planes, which can result in the formation of new active sites for the hydrogen evolution reaction (HER). Research data have shown that pure and Si-doped SrTiO_3_ have been experimentally synthesized.^20^ However, their photocatalytic ability to enhance water splitting and CO_2_ capture has yet to be reported, specifically in the context of nonmental/metalloid co-doping. Moreover, exploring the Si–As co-doped system of SrTiO_3_ will be highly significant for future researches in the field because integrated Si–As co-doping can increase the cleavage energies necessary for experimental exfoliation and scalable synthesis. Malathi *et al.* reported that co-doping significantly enhances the visible-light absorption and charge-separation efficiency of MoO_3_.^21^ By co-doping Si/As metalloids, there is also a high chance of band gap narrowing through the synergistic interactions between the Si 3p and As 4p orbitals, which passivates the trap state by single doping.

## 2 Research methods

The current work performed density functional theory (DFT) calculations based on the Quantum ESPRESSO approach to investigate the hydrogen evolution efficiency of SrTiO_3_ and its doped variants for visible light–driven photocatalytic water splitting.^22^ The geometric structure of pristine SrTiO_3_ was optimized according to experimental data obtained from the Materials Project repository.^23^ A plane-wave basis set and pseudopotential method were used to describe the ground-state properties of all systems. To obtain maximum stability, doping was performed in terms of ionic radius compatibility by replacing Ti atoms with either of Si or As atoms. Later, the systems were appropriately relaxed to the lowest ground-state energy till the energy on each atom became 0.02 eV per atom. Convergence was achieved using the Perdew–Burke–Ernzerhof (PBE) method within the generalized gradient approximation (GGA) framework.^24^ To achieve a balance between computational cost and accuracy, various convergence tests were performed with kinetic energy cut-off (ecut) values of 20, 30, 40, 50, 60, 70, and 80 Ry. Based on the convergence criterion, 60 Ry was chosen as the converged value of ecut together with a Monkhorst–Pack *k*-grid of 12 × 12 × 12. Norm-conserving pseudopotentials were used to accurately describe the valence electrons. To annihilate the underestimation of the band gap in standard DFT calculations and to ensure inclusion of d-orbitals of Sr and Ti atoms, we employed the LDA + *U* method with a Hubbard parameter.

The structural and dynamical stability of the systems was determined by the calculation of the formation energy of the systems based on the following equation.^25^1
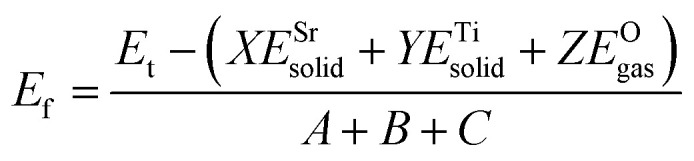
where *E*_t_ is the total energy of the SrTiO_3_ perovskite, *E*^Sr^_solid_, *E*^Ti^_solid_ and *ZE*^O^_gas_ denote individual energies of Sr and Ti atoms in their solid states and O atoms in their gaseous state, respectively. *A*, *B* and *C* represent the number of Sr, Ti and O atoms per unit cell, respectively. Further thermal and vibrational stability tests were performed by considering phonon frequency and molecular dynamics simulations, based on the equations2
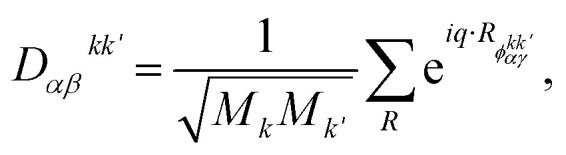
where *k* and *k*′ denote the atomic indices, and *α* and *β* represent Cartesian directions. *M*_*k*_ is the mass of the atom ‘*k*,’ and 
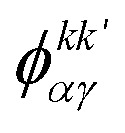
 is the interatomic force constant. The electronic interactions and orbital occupations were determined using the concept of the density of states (DOS) for a crystalline material, which is expressed as^26^3
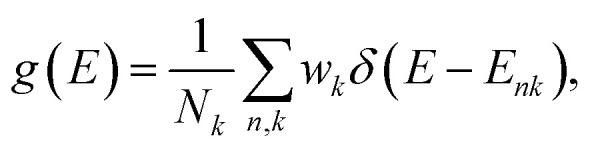
where *n* denotes the band index, and *k* represents *k*-points. With respect to photocatalytic behaviors, studies were performed on the current systems by considering the relation between the band gap and the overpotential required for the HER and OER based on the equation

4 *E*_e_ = *E*_b_ − 123 eV,

where *E*_b_ is the band gap in eV. Since SrTiO_3_ and its doped variants act as photocatalysts for splitting water into hydrogen and other constituents, it is necessary to consider the adsorption energy of water on the surface of these photocatalysts. Regarding this, we considered eqn (5) to calculate water adsorption energy (*E*_ad_).5
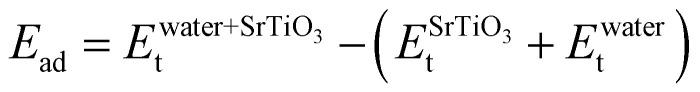
where 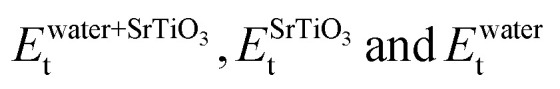 represent the total energies of the water-adsorbed photocatalyst, the photocatalyst alone and the water molecule alone, respectively. The optical responses of these photocatalysts to incident solar energy were evaluated using the following equation:

6 *ε*(*ω*) = *ε*_1_(*ω*) + *ε*_2_(*ω*),

where *ε*_1_(*ω*) and *ε*_2_(*ω*) are the real and imaginary parts of the dielectric function, respectively. The refractive index, which determines the ease of passage of the incoming photon.

## 3 Results and discussions

### 3.1 Structural and molecular dynamics analysis

**Fig. 1:**
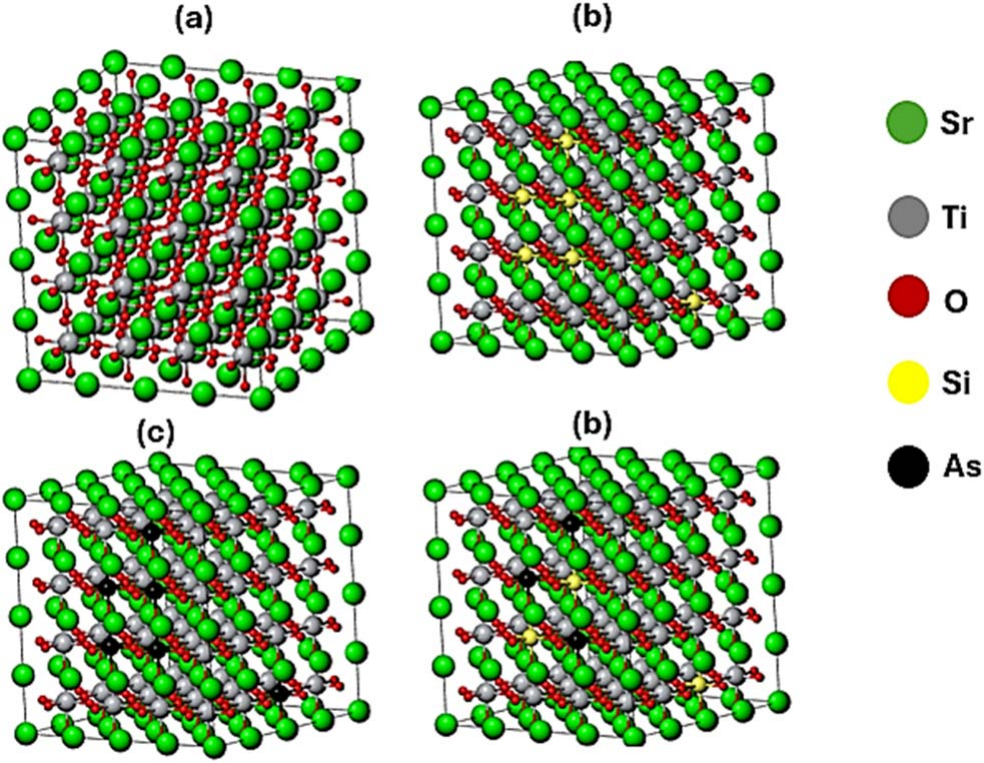
Optimized cubic system of the SrTiO_3_ perovskites: (a) pristine SrTiO_3_, (b) SrTiO_3_:Si (c) SrTiO_3_:As, and (d) SrTiO_3_:Si–As.

The pictorial structure of the systems under investigation is displayed in Fig. 1(a–d). During the process, pristine SrTiO_3_ underwent slight lattice distortions, leading to a series of bond length adjustments. For example, the Ti–O bond length was slightly reduced due to the introduction of Si impurities. As shown in Table 1, the optimized system of SrTiO_3_ exhibited highly stable behaviour due to a negative value of formation energy.^27^ Although Si^4+^ and Ti^4+^ are iso-valent, doping with Si resulted in a relatively complex structure because Si^4+^ has a smaller ionic radius than Ti^4+^ and Sr^4+^.^28^ So, energy did not converge directly when Si substituted Ti atoms. On this basis, the Si–Ti bonding process induced a significant lattice distortion. However, experimental reports have validated a stabilized Si-doped SrTiO_3_ (SrTiO_3_:Si) perovskite with a better charge compensation mechanism by creating oxygen vacancies (V_O) near Si sites.^29,30^ To align with experimental recommendations, we created an V_O near the Si site to annihilate the lattice distortion problem. Therefore, the electronic and other properties obtained for the SrTiO_3_:Si variant were based on the oxygen vacancy.

**Table 1:** Energy and structural stability parameters of SrTiO_3_ and its doped variants (X = Si and As)

Systems	Bond parameters			*E*_f_ (eV)	Water *E*_ad_ (eV)	Ref.
	Initial Ti–O bond (Å)	New Ti–O bond (Å)	New X–O bond (Å)			
SrTiO_3_	1.97	—	—	−3.58	−1.15	31
SrTiO_3_:Si	1.97	1.96	1.95	2.50	−1.30	32
SrTiO_3_:As	1.97	1.94	1.85	3.76	−0.99	Not reported
SrTiO_3_:Si–As	1.97	1.94	—	5.41	−1.57	Not reported

Furthermore, the negative formation energy obtained for pristine SrTiO_3_ indicates that SrTiO_3_ can be formed spontaneously under equilibrium conditions. Although positive values were obtained for Si- and As-doped system, they are regarded as stable because of the relatively small positive values. However, these systems require energy input for incorporation; hence, they cannot be formed spontaneously under equilibrium conditions. Their preparation involves the use of controlled conditions such as temperature, pressure and chemical environment. Among these two, SrTiO_3_:Si is the most stable because of its relatively lower value (2.50 eV). According to various experimental data, chemisorption of water molecules is strong on the SrTiO_3_ surface, forming hydroxyl groups.^33^ Therefore, we calculated water adsorption energies of pristine SrTiO_3_ and its doped variants. By considering the 001 surface of SrTiO_3_, adsorption energies of −1.15, −1.30, −0.99 and −1.57 eV per H_2_O molecule were obtained for SrTiO_3_, SrTiO_3_:Si, SrTiO_3_:As, and SrTiO_3_:Si–As, respectively. The obtained values fall within the −0.8 to −2.0 eV range as reported by others.^34^

**Fig. 2:**
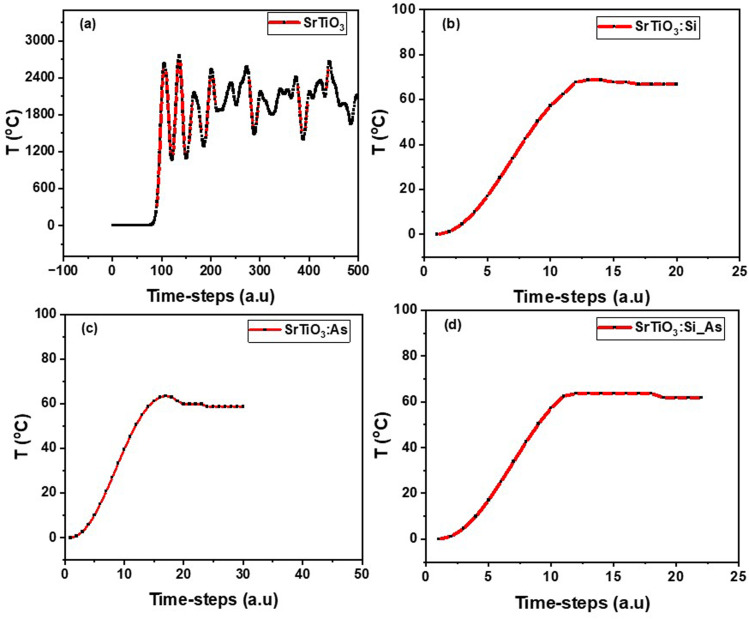
Temperature dynamics of the SrTiO_3_ perovskite and its doped species (a) pristine SrTiO_3_, (b) SrTiO_3_:Si (c) SrTiO_3_:As, and (d) SrTiO_3_:Si–As.

Mechanistically, water splitting on the perovskite surface is a finite-temperature, dynamic phenomenon, which involves fluctuations in hydrogen bonds.^35^ It also involves thermally activated proton transfer, lattice vibrations and surface reconstruction.^36^ Since ground-state DFT alone deals with a single configuration, it is necessary to employ molecular dynamics analysis to obtain a realistic ensemble of molecular states for an accurate description of adsorption, dissociation and radical reaction pathways.^37,38^ On this basis, we study the molecular dynamics of the systems under investigation (Fig. 2(a–d)). As shown in Fig. 2(a), at the ground temperature (0 °C), the SrTiO_3_ system was initially at rest at a very low energy state. A sudden indication of energy input emerged due to a sharp temperature rise of 200 K in the first 100-time steps. It also indicates thermal excitation in SrTiO_3_ due to UV interactions. The temperature continues to fluctuate between 1500 °C and 2500 °C, signifying that SrTiO_3_ is dynamically stable at high temperatures. However, the energy exchange among atoms stabilizes at this point but still allows for some thermal motion. The ability of SrTiO_3_ to endure high temperatures without collapsing validates its photocatalytic behaviours under UV irradiation. However, consistent fluctuations at high temperatures indicate poor thermal management due to insufficient charge carriers.^39^ Regarding this, its photocatalytic performance is expected to be limited. Based on previous data, doped variants of SrTiO_3_ undergo photocatalysis at conditions slightly higher than room temperature.^40^ Considering Si-doping (Fig. 2(b)), temperature stabilized quickly around 68 °C in a short number of time steps, in accordance with the optimum temperature range reported for visible light photocatalysis (25 °C–80 °C).^41^ The temperature of the As-doped system (Fig. 2(c)) stabilized after a longer time (50 time steps) than that of the Si-doped system, indicating a relatively higher thermal energy contribution. The shortest and potentially fastest stabilization was observed for the Si–As co-doped system (Fig. 2(d)), offering the highest efficiency. Notably, the corresponding temperatures of the Si-doped, As-doped and Si–As co-doped systems were 68.85 °C, 61.94 °C and 61.40 °C, respectively. In addition to stabilizing at the lowest temperature, Si–As co-doped SrTiO_3_ transitioned to another lower temperature after 18 time steps, indicating its ability to stabilize in various temperature phases. Based on the molecular dynamics simulations, pristine SrTiO_3_ is likely inefficient because of its consistent temperature instability. In contrast, Si- and As-doped systems demonstrate improved efficiency because of better thermal properties, while the Si–As co-doped system is potentially appropriate for efficient hydrogen production applications.

### 3.2 Vibrational (phonon) and tensile properties

**Fig. 3:**
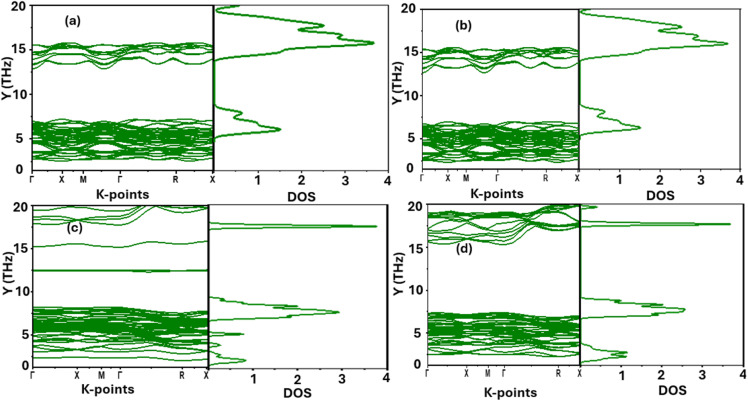
Phonon dispersion curves and phonon DOS of the SrTiO_3_ perovskite and its doped variants: (a) pristine SrTiO_3_, (b) SrTiO_3_:Si (c) SrTiO_3_:As, and (d) SrTiO_3_:Si–As.

In photocatalysis, mechanically stable materials are required for long-term operation under harsh conditions (such as thermal expansions, acidic or alkaline environments). Thus, phonon dispersion analysis is necessary to confirm the dynamic stability of the investigated structures. Moreover, conducting phonon analysis can help address some problems related to overheating in SrTiO_3_.^42^ The phonon dispersion curves of pure to co-doped SrTiO_3_ variants are displaced in Fig. 3(a–d). The force constants were calculated at 0.01 Å for each atomic displacement along the direction of the lattice vectors. As observed, all systems revealed positive frequency throughout the Brillouin zone, in agreement with a previous report.^43^ Through this, the systems are said to be stable under vibrational frequencies. The absence of imaginary frequencies suggests no dynamic instability related to any of these systems. In all systems, the active vibrational modes can be seen corresponding to various DOS peaks. Other broad vibrational modes can also be seen, confirming the stability of these systems for further investigations.

**Fig. 4:**
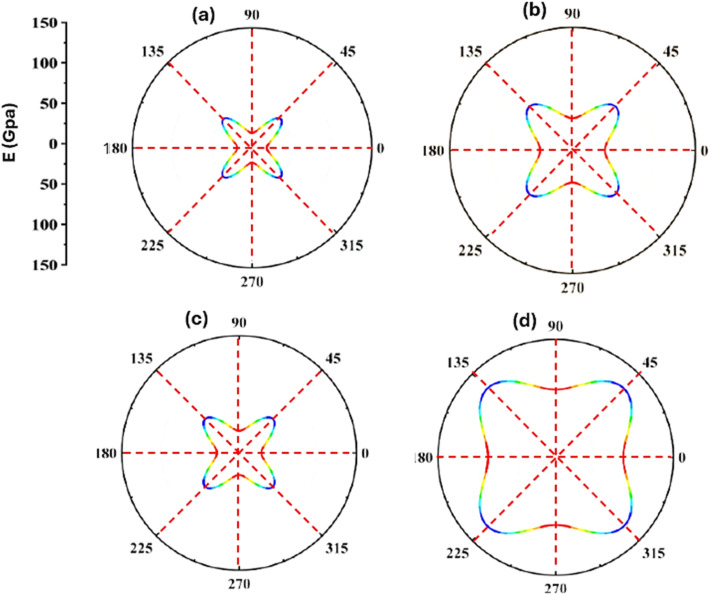
Young's modulus orientation of the SrTiO_3_ perovskites: (a) pristine SrTiO_3_, (b) SrTiO_3_:Si (c) SrTiO_3_:As, and (d) SrTiO_3_:Si–As.

A previous report has confirmed that the catalytic activity of SrTiO_3_ is strongly strain dependent.^44^ For example, Linyuan *et al.* reported a strain-dependent oxygen evolution reaction on the SrTiO_3_ surface.^45^ Additionally, water adsorption, dissociation and proton transfer create some local lattice distortions.^46^ Evaluating elastic constants is essential for verifying the mechanical stability of the DFT-optimized structures, which allows the assessment of deformation properties under certain photocatalytic operating conditions. Analysing tensile properties also paves the way for highlighting strain-induced modifications in the electronic structure and reaction energetics. In this case, we proceed with an investigation of the tensile properties of the current systems by considering Young's moduli and Poisson's ratio. As summarized in Table 2, Young's modulus data revealed symmetric behaviors in SrTiO_3_ and its various doped variants. The SrTiO_3_:Si–As variant exhibits the highest value of 159.43 GPa, indicating its high resistance to mechanical deformation. A high Young's modulus is crucial for the HER because it suppresses lattice deformation during hydrogen adsorption/desorption, stabilizes catalytic active sites, enhances electron transfer by reducing vibrational scattering, and improves mechanical durability under operational stress. This results in consistent Δ*G*_H, robust catalytic performance, and improved long-term HER stability. The calculated Young's modulus values for pristine SrTiO_3_, SrTiO_3_:Si, and SrTiO_3_:As were 53.10, 76.29 and 72.41 GPa, respectively. Therefore, doping the pure system of SrTiO_3_ improves its mechanical strength, which is crucial to various optoelectronic applications. As shown in Fig. 4(a–d), values of Young's moduli for these systems attained a maximum when stress was applied at 90° relative to the frame of reference.

**Table 2:** Tensile parameters

Systems	Minimum values		Maximum values	
	*Y*_min_ (Gpa)	*V*_min_	*Y*_max_ (GPa)	*V*_max_
SrTiO_3_	20.03	0.185	53.10	0.772
SrTiO_3_:Si	32.00	0.172	76.29	0.541
SrTiO_3_:As	37.79	0.176	72.41	0.660
SrTiO_3_:Si–As	78.22	0.153	159.43	0.411

**Fig. 5:**
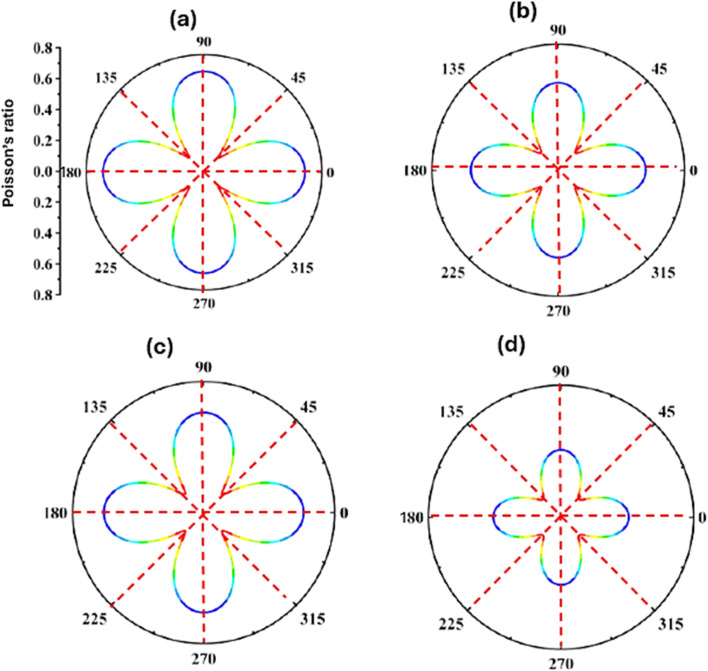
Poisson's ratio orientation of the SrTiO_3_ perovskites: (a) pristine SrTiO_3_, (b) SrTiO_3_:Si (c) SrTiO_3_:As, and (d) SrTiO_3_:Si–As.

To investigate the effects of unidirectional stress, we considered Poisson's ratio analysis. It represents the response of materials based on transverse and longitudinal strain and has no units. The calculated Poisson's ratio for the current systems ranged from 0.153 to 0.772, with the SrTiO_3_:Si–As having the lowest value. Based on the positive values obtained, dimensions of these materials increase when compressed and then decrease when stretched. As shown in Fig. 5(a–d), the maximum values were attained at 90°, while minimum values were attained at 45°.

### 3.3 Electronic band gap and band alignment

The electronic properties of photocatalysts are primary parameters that expose their physical ability to enhance the two key photocatalytic reactions: the hydrogen evolution reaction (HER) and oxygen evolution reaction (OER).^47^ Mechanistically, the valence band is the region where the OER takes place *via* removal of an electron driven by incident light energy.^48^ On the other hand, the HER takes place within the conduction band through the transfer of photogenerated electrons from the CB to protons. These two key processes occur according to equations,^49^

7 4H^+^ + 4e^−^ → 2H_2_

8 2H_2_O → O_2_ + 4H^+^ + 4e.

**Fig. 6:**
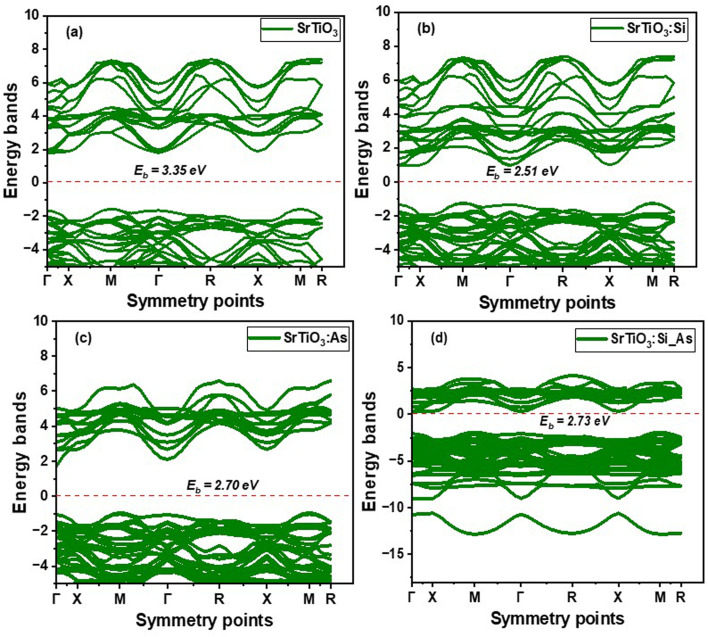
Electronic band diagrams of (a) pristine SrTiO_3_, (b) SrTiO_3_:Si (c) SrTiO_3_:As, and (d) SrTiO_3_:Si–As. Each of the perovskite configurations revealed typical semiconductor properties.

For water molecules to be fully decomposed and yield hydrogen, the band edge values for the HER and OER must be greater than or equal to 0 eV and 1.23 eV, respectively. In other words, both valence band maximum (VBM) and conduction band minimum (CBM) must approach the Fermi energy level. In this work, we studied the electronic band structures of four different configurations of the SrTiO_3_ perovskites.^50^ Furthermore, the electronic density of states (DOS) and partial density of states (PDOS) were calculated to understand the contributions of various electronic states and orbitals to the photocatalytic performance of these systems. As shown in the band structure diagrams in Fig. 6(a–d), pristine SrTiO_3_ demonstrates a wide indirect band gap of 3.35 eV, in good agreement with previous experimental data.^51^ However, its efficiency would be limited due to absorption of ultraviolet (UV) under solar illumination.^52^ Conversely, SrTiO_3_:Si exhibits significant band narrowing to the value of 2.31 eV, applicable for visible-light absorption, which accounts for the highest percentage of the solar spectrum. The band-gap narrowing is due to the introduction of some new energy states near the conduction band, enhancing carrier mobility. Moreover, the band gap in SrTiO_3_:Si is narrowed because V_O introduces donor-like energy states within the band gap, which paves the way for interband transitions. Another reason is that Si atoms create localized states near the conduction band, enhancing conductivity.

In relation to the band gap, the overpotential region represents the vicinity of the band gap above 1.23 eV necessary for water splitting. It is represented by the equation^53^

**Fig. 7:**
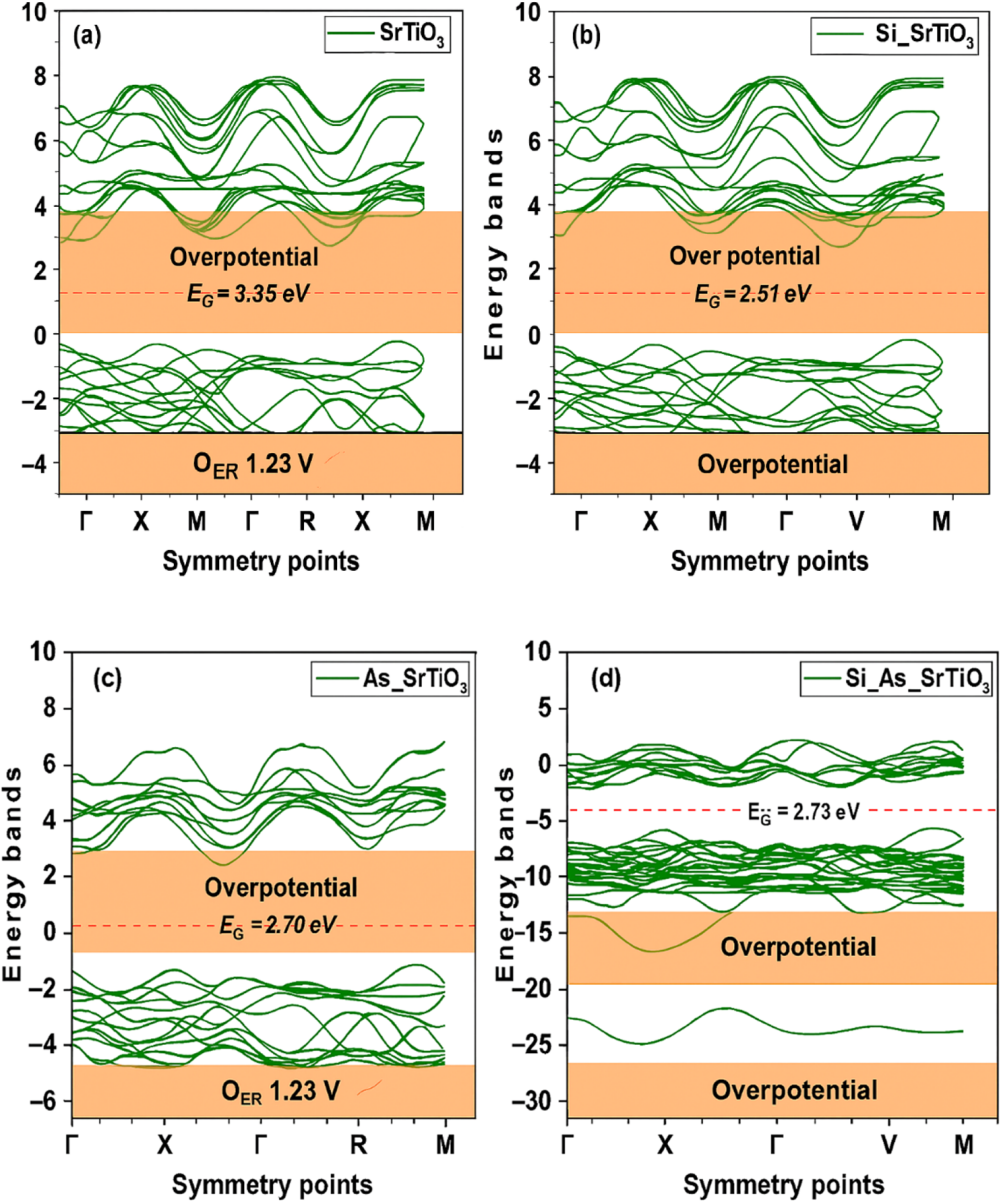
Overlay potential regions displaying the redox potentials for the HER and OER. (a) Pristine SrTiO_3_, (b) SrTiO_3_:Si (c) SrTiO_3_:As, and (d) SrTiO_3_:Si–As.

9 *E*_p_ = *E*_b_ − 1.23 eV,

where *E*_b_ is the band gap in eV. Based on eqn (9), the calculated overpotential values in the order of pure > SrTiO_3_:Si > SrTiO_3_:As > SrTiO_3_:Si–As are 2.12, 1.28, 1.47 and 1.50 eV, respectively. Although pristine SrTiO_3_ possesses sufficient *E*_p_, it has not been utilized efficiently due to excessive UV absorption. On the other hand, the smaller *E*_p_ exhibited by the Si-doped system is considered more efficient because of better visible light absorption. In the context of hydrogen evolution, these overpotential values represent excess energy that can improve photocatalytic reaction kinetics and can be lost as heat if not fully utilized. The effects of doping with As atoms are shown in Fig. 6(c). The band gap of SrTiO_3_:As is wider than that of SrTiO_3_:Si because As has additional 4p orbitals, which do not contribute substantially to the conduction band. Instead of this, they tend to push the valence band down, resulting in a wider band gap. With proper band alignment, the overpotential (1.47 eV) obtained for SrTiO_3_:As can drive efficient water splitting. Overall, the narrowest overpotential offered the best photocatalytic performance because it presents the smallest heat energy loss, which can overcome kinetic barriers associated with the OER.^54^ In all cases, the overpotential values are sufficient to straddle efficient HER and OER, with SrTiO_3_:Si–As showing promising efficiency. The overlay band diagrams displaying typical alignment with the CBM (for the HER) and VBM (for the OER) are shown in Fig. 7(a–d). In each case, the redox potentials are highlighted to depict band edge alignment and quantify the energy needed or available to drive these reactions.

**Fig. 8:**
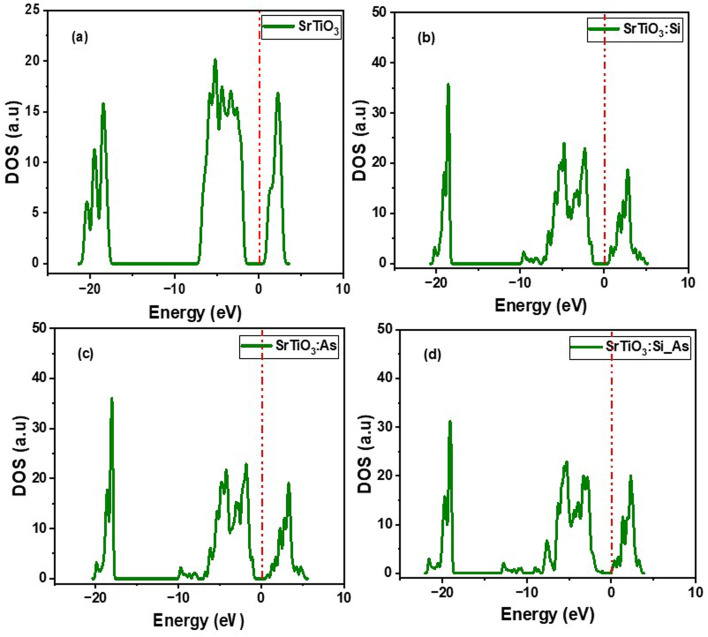
DOS diagrams of (a) pristine SrTiO_3_, (b) SrTiO_3_:Si (c) SrTiO_3_:As, and (d) SrTiO_3_:Si–As. Flat energy states at the Fermi level in each of the perovskite configurations revealed typical semiconductor properties.

**Fig. 9:**
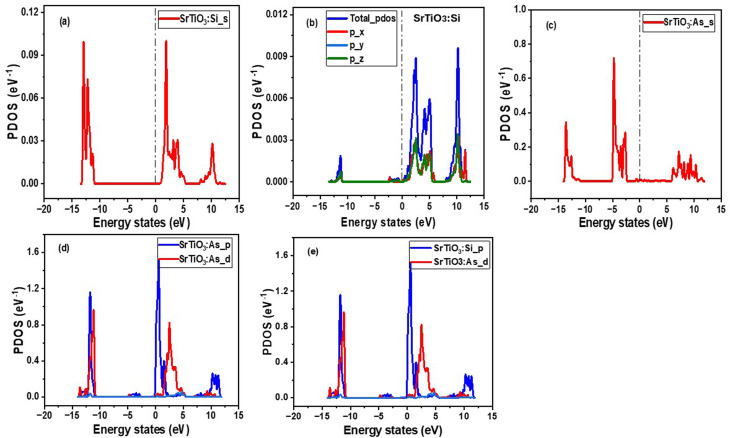
Various orbital contributions in the doped variants of the SrTiO_3_ photocatalyst in the context of water splitting: (a) s orbitals in SrTiO_3_:Si, (b) p orbitals in SrTiO_3_:Si, (c) s orbitals in SrTiO_3_:As, (d) p and d orbitals in SrTiO_3_:As, and (e) s, p and d orbitals in SrTiO_3_:Si–As.

The DOS diagrams are shown in Fig. 8(a–d). Fig. 8(a) shows that Si impurities introduce mid-gap states (near −4.4 eV), which actively took part in narrowing the wide band gap of SrTiO_3_. Similarly, additional mid-gap states (near −4 eV) can be seen in the SrTiO_3_:As. As co-doping took place, a systematic redistribution of the DOS is observed near the Fermi level. In all four systems, more energy states appeared in the valence band than in the conduction band. An increase in intensity of the DOS peaks in the doped SrTiO_3_ species can be attributed to several electronic and structural mechanisms, which include new impurity states, localized states and modified charge carrier dynamics. Based on the characteristics of the PDOS pattern displayed in Fig. 9(a–e), Si doping introduces donor-like states in SrTiO_3_ due to s-orbital contribution near the edge of the conduction band, leading to improved electron transfer. We also observed the strongest contribution from the p^y^ orbital, leading to 2p hybridization in O. In the SrTiO_3_:As, s orbitals introduced localized energy states near the valence band, which suppresses anticipated charge-carrier recombination risks. However, effects of recombination are reduced by the presence of p and d orbitals near band edges of SrTiO_3_:As. There are synergetic contributions from p orbitals of Si and As and d orbitals of Ti, leading to synergistic modification with improved charge dynamics. The summarized orbital contributions and the role they play in the photocatalytic hydrogen evolution are summarized in Table 3.

**Table 3:** Summary of the observed orbital features and their role in enhancing the water-splitting process in doped SrTiO_3_ variants

Orbitals in materials	Contributing orbitals	Activity in H_2_ evolution
s orbital in SrTiO_3_:Si	s orbital of Si	Narrows band gap and improves the HER
p orbital in SrTiO_3_:Si	p orbital of Si/O	Improves charge separation and the OER
s orbital in SrTiO_3_:As	s orbital of As	Enhances visible light absorption
p and d orbitals in SrTiO_3_:As	s orbital of As and d orbital of Ti	Supports electronic transport and the HER/OER
s orbital in SrTiO_3_:Si–As	s orbital of Si/As and d orbital of Ti	Provides synergic balance, strong absorption, efficient HER and OER

### 3.4 Electron localization function (ELF)

**Fig. 10:**
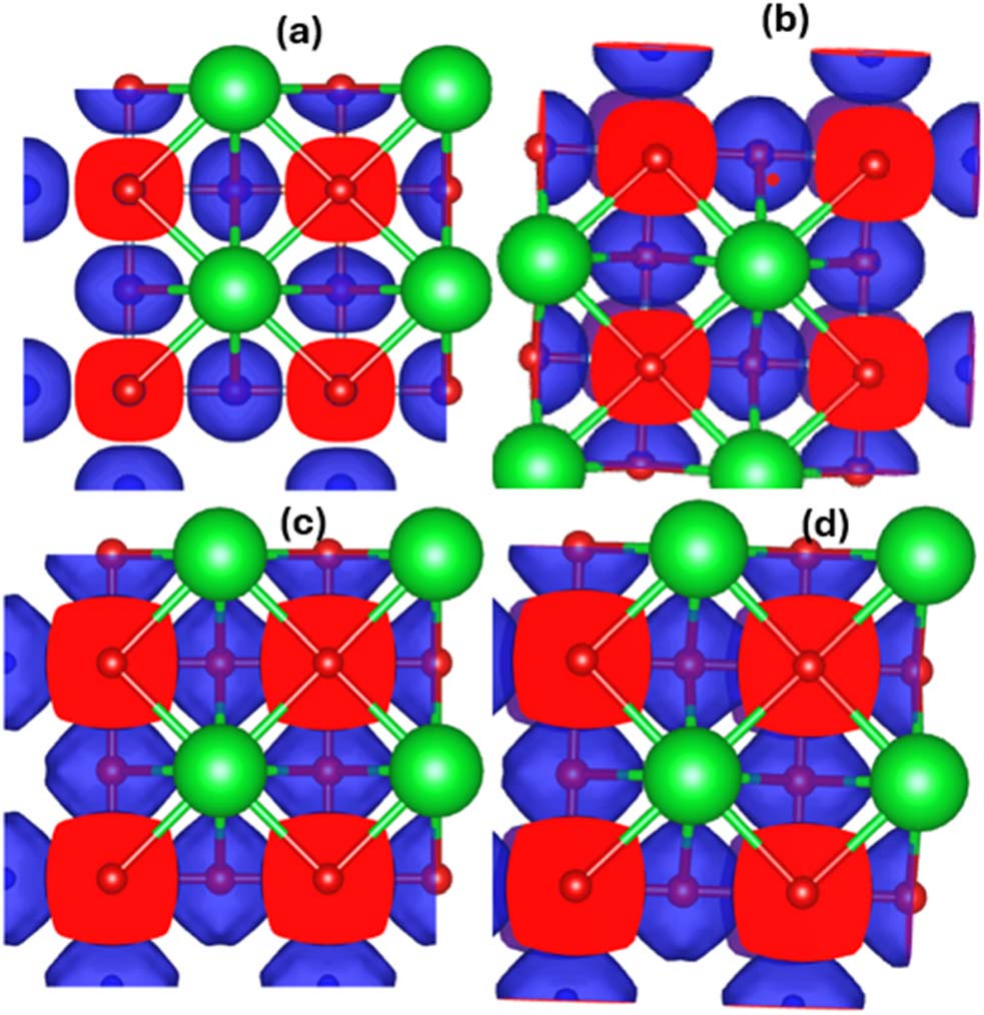
ELF images of the SrTiO_3_ photocatalyst and its doped variants. (a) Pristine SrTiO_3_, (b) SrTiO_3_:Si (c) SrTiO_3_:As, and (d) SrTiO_3_:Si–As.

The concept of electron localization function (ELF) plays big role in understanding the photocatalytic water-splitting phenomena. This is achieved by understanding how electrons are dispersed within the structure of a photocatalyst.^55^ Properties such as chemical bonding characteristics are identified by ELF analysis.^56^ Regarding water splitting, catalyst surface-water interactions are strongly influenced by bonding characteristics. A previous report has confirmed that localized electrons residing near the catalyst surface enhance the adsorption and activation of reactive radicals.^57^ ELF images obtained for the current systems are shown in Fig. 10(a–d).

For pristine SrTiO_3_, two regions of high ELF (red zones) and moderate ELF (blue zones) can be seen. In the high ELF region, strong electron localization is seen near oxygen, indicating the presence of lone pairs. The presence of these lone pairs also describes the covalent character of the Ti–O bond. In the moderate region, bonding between Ti and O atoms occurs due to the mixed ionic-covalent nature of the Ti–O bond. Typically, Sr atoms (green) demonstrate low ELF, resulting in delocalized electrons. Therefore, Sr behaves as an electron donor, contributing to the ionic behaviour of the lattice without forming strong directional bonds. Overall, chemical bonds in SrTiO_3_ are not purely ionic, and oxygen significantly plays a major role in electron localization, which is crucial for properties such as dielectric behaviour, ferroelectricity, and photocatalytic activity.^58^ In Fig. 10(b), Si impurities introduce distortions around the Si sites, which indicates modified bonding environments. The localized states near Si trap and guide electrons and help enhance charge separation with a reduced rate of recombination, which improves the HER process. With As impurities (Fig. 10(c)), the ELF is more symmetric, specifically around As atoms. Due to this, there are stronger polarizations and a probability of directional charge movement, which is highly significant for the OER. Interestingly, the idea of co-doping (Fig. 10(d)) with Si and As atoms leads to a more complex ELF pattern with better localization and symmetry. The synergistic effects between Si and As impurities improve both electron and hole mobility. Together, these behaviours provide optimal conditions for efficient overall water splitting. The overall ELF observations reveal that doping SrTiO_3_ with these atoms significantly alters its electronic structure and charge dynamics.

### 3.5 Optical spectra

**Fig. 11:**
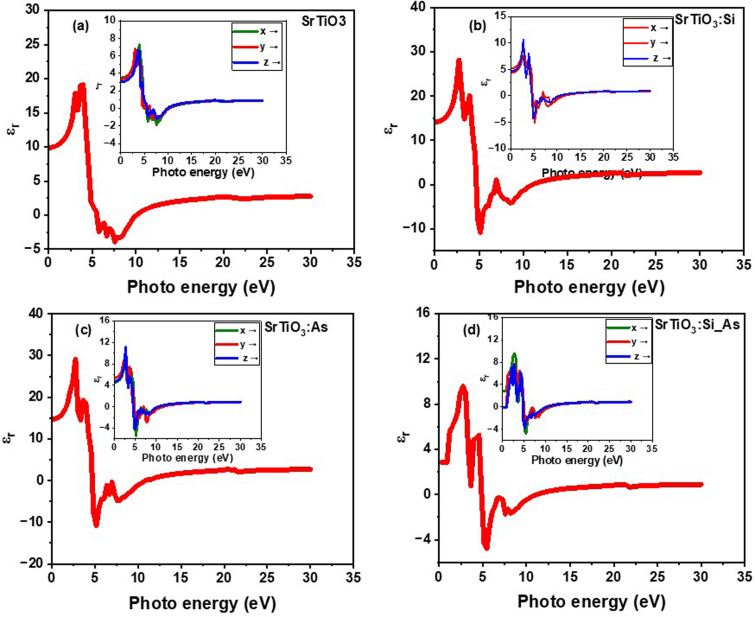
Real dielectric spectra of pristine SrTiO_3_ and its doped variants. (a) Pristine SrTiO_3_, (b) SrTiO_3_:Si (c) SrTiO_3_:As, and (d) SrTiO_3_:Si–As. All the corresponding insets reveal the characteristic real dielectric features along various anisotropic directions.

Photocatalytic water splitting largely depends on photon absorption, which excites electrons across the Fermi level. The process generates electron–hole pairs that drive both the HER and OER. According to widely accepted literature, the optical response of materials strongly depends on their dielectric functions. Diagrams in Fig. 11 display the real dielectric response of pristine SrTiO_3_ and its doped variants as a function of photon energy. Various subfigures correspond to different materials, and the insets display anisotropic features along *x*, y and *z* crystallographic directions.

The peaks in the real part of the dielectric function represent strong interband transitions, which are necessary for effective light harvesting. As shown in Fig. 11(a–d), all spectra reveal anisotropic features, as observed in the insets. With anisotropic features, charge carrier mobility and separation can be influenced by homogeneity. Generally, the diagrams reveal how impurities modify the optical behaviours of SrTiO_3_ for efficient water splitting under solar irradiation. Fig. 11(a) shows significant activity of the real dielectric largely in the UV region, consistent with an optical band gap of 3.2 eV. Upon Si and As doping and As–Si co-doping, we observed new features especially at lower energy levels. The observed shifts indicate band-gap narrowing and improved photon absorption in the visible-light range, which are essential for efficient solar-to-hydrogen conversion. Among the investigated systems, the broadest and most intense response corresponds to SrTiO_3_:Si–As, indicating superior light harvesting for improved photocatalysis.

**Fig. 12:**
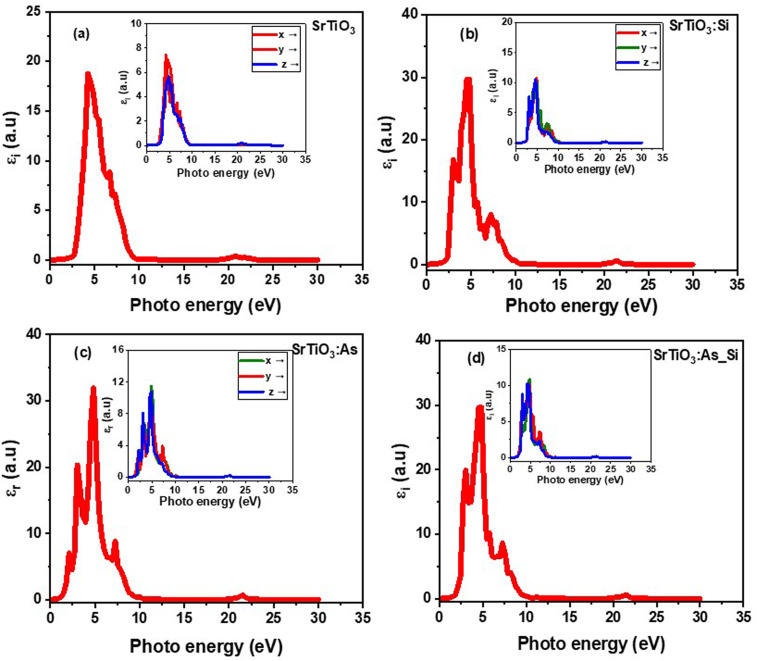
Imaginary dielectric spectra of pristine SrTiO_3_ and its doped variants. (a) Pristine SrTiO_3_, (b) SrTiO_3_:Si (c) SrTiO_3_:As, and (d) SrTiO_3_:Si–As. All the corresponding insets show the characteristic imaginary dielectric features along various anisotropic directions.

**Fig. 13:**
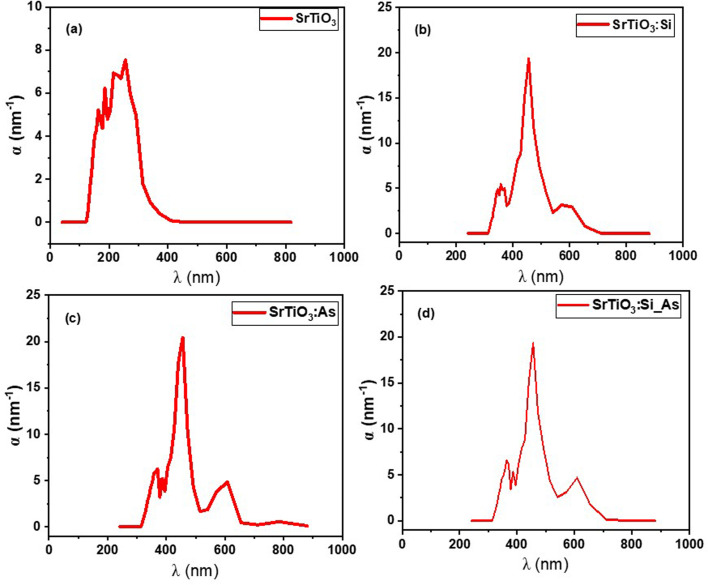
Optical absorption coefficient spectra of the pure and doped species of the SrTiO_3_ photocatalysts. Each spectrum indicates the range of visible light that the compounds can absorb. (a) Pristine SrTiO_3_, (b) SrTiO_3_:Si (c) SrTiO_3_:As, and (d) SrTiO_3_:Si–As.

Fig. 12(a–d) depict the imaginary components of the dielectric functions for pristine SrTiO_3_ and its corresponding doped species. The imaginary dielectric functions vary directly with a critical parameter for photocatalytic water splitting known as the optical absorption.^59^ This is because the imaginary dielectric function determines the efficiency of solar energy absorption and generation of charge carriers.^60^ In Fig. 12(a), strong absorption in the UV region was observed because of the loss extension to 3.3 eV, which is consistent with the SrTiO_3_ band gap. Therefore, the optical behaviour of pristine SrTiO_3_ limits its ability to photocatalyze water splitting under visible light. The corresponding inset also indicates homogeneous optical response without directional charge separation. After introducing Si (Fig. 12(b)), we observed new characteristic peaks at lower photon energies, which enhanced band gap narrowing to the visible range. The corresponding inset indicates anisotropy, which prompts precise separation of reactive radicals and reduced recombination of radicals. Activities of As impurities in pristine SrTiO_3_ (Fig. 12(c)) produce strong interband transitions with better anisotropy along specific crystallographic directions, which is highly significant for the OER. The synergetic effects of As–Si co-doping (Fig. 12(d)) produce the most intense imaginary peak in the visible-light range, which translates to superior light absorption. The characteristic feature of the SrTiO_3_:Si–As system favours both the HER and OER. Overall, the imaginary spectra reveal how Si and As impurities efficiently tune the optical properties of SrTiO_3_ to strong visible-light absorption with improved charge separation and reduced recombination rates. Various spectra of the optical absorption coefficient are shown in Fig. 13(a–d). Pristine SrTiO_3_ exhibits strong UV absorption and very low visible-light absorption (<10 nm^−1^), while SrTiO_3_:Si, SrTiO_3_:As and SrTiO_3_:Si–As variants show improved absorption as high as 25 nm^−1^, specifically in the visible-light range (400–800 nm) because of induced band-gap narrowing. Si doping improves light harvesting *via* conduction-band modulation, which enhances the HER, while As doping introduces intermediate states enhancing carrier mobility, which improves the OER. The SrTiO_3_:Si–As system shows the broadest and strongest visible-light absorption, suggesting synergistic effects that optimize electronic transitions and photocatalytic efficiency for solar-driven water splitting. The summarized optical absorption properties are thus displayed in Table 4. In Table 5, we compare the calculated properties of the present systems with those of some previously reported systems. Notably, most of the parameters reported in the current study aligned with previously reported values.

**Table 4:** Visible-light absorption range and water-splitting potentials of the current systems

SrTiO_3_ species	Band gap properties	Absorption range (nm)	Ability to interact with visible light	Water splitting potentials
Pristine SrTiO_3_	Wide (3.2 eV)	200–300 (UV)	Relatively poor	Low
SrTiO_3_:Si	Narrowed	300–550 (UV-vis)	Improved mobility	Moderate
SrTiO_3_:As	Narrowed	300–570	Enhanced light harvesting	High
SrTiO_3_:Si–As	Significantly tuned	300–570	Synergistic doping and better absorption profile	Very high

**Table 5:** Comparison of the present results with those of the previously reported works

Systems	*E*_g_ (eV)	Absorption range (nm)	Interaction with visible light	Efficiency	Similar result from previous works	Ref.
Pristine SrTiO_3_	3.35	200–300 (UV)	Poor	Low	Undoped SrTiO_3_ with a Rh cocatalyst (band gap of 3.3 eV and absorption of 290 nm), presenting strong UV behaviours	61
SrTiO_3_:Si	2.51	300–550 (UV-vis)	Improved	Moderate	Si-doped SrTiO_3_ (DFT study on site substitution). Band gap of 2.4 eV and visible light absorption in the 300–500 nm region	62
SrTiO_3_:As	2.70	300–570	Enhanced light harvesting	High	Sb-doped SrTiO_3_ with a RhCrOx cocatalyst (absorption 40–700 nm), demonstrating good visible-light harvesting	63
SrTiO_3_:Si–As	2.73	300–570	Synergistic doping effects	Very high	Al–Ce-co-doped BaTiO_3_ nanofibers with piezo-potential-enhanced photocatalytic properties	64

## 4 Conclusion

Hydrogen fuel produced *via* photocatalytic water splitting is one of the greenest renewable energies for better efficiency.^65^ The present study investigated the significance of nonmetal/metalloid co-doping in the context of water splitting using strontium titanate. Based on our investigation into SrTiO_3_ and its doped derivatives, all the characteristic features observed for pristine SrTiO_3_ confirmed its full interaction with UV photons, which required very high temperatures for water splitting. However, doping precisely improves the ‘suitability of the material for photocatalytic water splitting. For example, temperature variations revealed that undoped SrTiO_3_ exhibited poor thermal regulation and high overpotential, limiting its efficiency. By doping with Si, SrTiO_3_ experienced rapid thermal stabilization in the optimal photocatalytic temperature range. The Si–As co-doped system demonstrated better efficiency across all evaluated parameters, including the fastest thermal stabilization, the highest mechanical strength (159.43 GPa), and the lowest overpotential, indicating enhanced charge-carrier dynamics and structural resilience. Phonon dispersion analysis confirmed the dynamic stability of all systems, and band structure analysis revealed band gap narrowing due to the introduction of new energy states near the conduction band, facilitating improved carrier mobility. Based on optical spectra, all doped variants of SrTiO_3_ exhibit good interactions with visible light for efficient HER and OER. These findings highlight the synergistic advantages of co-doping in optimizing SrTiO_3_ for efficient, stable, and scalable water splitting applications.

## Consent for publication

All authors of this work have agreed and are ready to sign the Transfer of Copyright, which empowers the Publisher to protect the work against unauthorized use and to maintain the integrity of the work from a bibliographical and archival standpoint.

## Author contributions

Conceptualization, formal investigation and writing – original draft: Y. S. I. methodology, project administration, review and validation: Y. S. I. and M. U. K. Supervision, review, software and resources: M. U. K. and F. B.

## Conflicts of interest

The authors declare that they have no known competing financial interests or personal relationships that could have appeared to influence the work reported in this paper.

## Data Availability

The code for Quantum ESPRESSO used to generate new data for this manuscript can be found at https://www.quantum-espresso.org/. The version of the code employed for this study is version 7.4. Numerical data for some electronic properties' calculations are contained within the manuscript, while other data sets are not human readable.
